# Perceptual shrinkage of a one-way motion path with high-speed motion

**DOI:** 10.1038/srep30592

**Published:** 2016-07-28

**Authors:** Yutaka Nakajima, Yutaka Sakaguchi

**Affiliations:** 1 Laboratory for Human Informatics, Graduate School of Information Systems, The University of Electro-Communications, Tokyo, Japan.

## Abstract

Back-and-forth motion induces perceptual shrinkage of the motion path, but such shrinkage is hardly perceived for one-way motion. If the shrinkage is caused by temporal averaging of stimulus position around the endpoints, it should also be induced for one-way motion at higher motion speeds. In psychophysical experiments with a high-speed projector, we tested this conjecture for a one-way motion stimulus at various speeds (4–100 deg/s) along a straight path. Results showed that perceptual shrinkage of the motion path was robustly observed in higher-speed motion (faster than 66.7 deg/s). In addition, the amount of the forwards shift at the onset position was larger than that of the backwards shift at the offset position. These results demonstrate that high-speed motion can induce shrinkage, even for a one-way motion path. This can be explained by the view that perceptual position is represented by the integration of the temporal average of instantaneous position and the motion representation.

In the field of visual psychophysics, position coding of moving objects has long been one of the essential research topics. It is a long-standing assumption that position information and motion information are processed separately in our visual system^1^,^2^. However, a large number of studies have shown that motion can affect the perception of position^3^: as a sustained shift of a window in which carrier motion is embedded^4^,^5^,^6^,^7^,^8^, as a shift associated with transiency^9^,^10^,^11^,^12^,^13^,^14^,^15^,^16^, as a shift caused by motion adaptation^17^,^18^, as a shift of offset position of a moving object, i.e. a representational momentum^19^,^20^,^21^, and as a mislocalisation of the path of a moving object, including onset and/or offset positions^22^,^23^,^24^,^25^,^26^,^27^,^28^,^29^,^30^,^31^,^32^,^33^.

The position of a one-way moving object is generally perceived shifted toward the motion direction. A representative example is the “flash-lag effect”^9^,^13^,^14^,^15^: a moving object is perceived being ahead of a briefly flashed object when the two are physically aligned. Another example is the “Fröhlich effect,”^34^,^35^,^36^ where the onset position of a moving object is perceived shifted in the motion direction if the moving object appears abruptly. In addition, forwards displacement is observed when another object moving in parallel suddenly disappears^12^.

Another type of perceptual positional shift is the shrinkage of a moving path, which is typically observed for back-and-forth motion^15^,^30^. When an object goes along a straight path in the reciprocating manner at a constant speed or with a sinusoidal velocity, the perceived endpoints are shifted toward the centre of the path. This shrinkage could be explained by the integration of spatiotemporal information around the endpoints if we consider a mechanism of the type shown in Fig. 1. In this figure, the slope of the black diagonal line represents the velocity of the object. Though both positional and motion information should be integrated around the endpoint, the motion vectors are cancelled out (or temporally smoothed) because the motion direction is reversed, and thus, only the positional information would be integrated within a fixed temporal window (less than 1 second)^30^. Consequently, the endpoint is perceived simply as the spatially averaged position within the temporal window. In the case of reciprocating motion (with cancelling out of the motion signal at the endpoints), the amount of shrinkage is proportional to the motion speed: faster motion around the endpoint induces larger shrinkage of the path, and vice versa. The model can also explain the finding that the shrinkage hardly occurs when a 1-second standstill (pause) is inserted before the direction reversal^30^, because the result of averaging within a short (less than 1 second) temporal range should be the same as when the motion continues. This averaging view is consistent with the postdictive hypothesis that explains the positional shift of a moving object accompanied by flash stimuli^11^,^15^.

However, the temporal averaging model seems to remain controversial as an explanation for the position perception of objects on a one-way motion path. Some studies have shown that one-way motion (speed: 8.0 deg/s) did not cause perceptual shrinkage; rather it induced a little overshoot (i.e. perceptual elongation of the path)^30^,^33^, contradictory to the temporal averaging view that leads to a forwards perceptual shift of position at motion onset and/or a backwards shift at motion offset (Fig. 1). Nevertheless, when we carefully apply this view to one-way motion, it can explain why shrinkage is not observed for this type of motion. Figure 2a depicts the spatiotemporal components contained in one-way motion at the offset position (the case of the onset position can be explained in the same manner). When the instantaneous position is averaged within a temporal window, the averaged position does not deviate much from the veridical offset position, so long as the motion speed is slow; thus, the perceptual shrinkage and/or shift would not occur or be very small (Fig. 2a). In contrast, when the object moves at a higher speed, the averaged position at the offset is further from the veridical offset position (Fig. 2b). Following this model, one can reasonably assume that relatively slow one-way motion induces only a small amount of perceptual shift. This in turn means that the one-way stimulus at 8.0 deg/s^30^ does not satisfy sufficient conditions to induce shrinkage of the one-way path. We can expect, therefore, that the onset (offset) position must be perceived as shifted forwards (backwards) when the motion speed is sufficiently fast.

The temporal averaging view, as described above, successfully explains the perceptual endpoint of one-way moving objects, as well as the endpoints of reciprocating motion^30^ and their speed dependency. Note that we have not taken account of the motion representation (assuming that there are no motion signals at the endpoint) in the above discussion. Therefore, our next question is how motion representation (i.e. motion momentum) would affect the perceptual shift. Considering that motion momentum is observed at the offset position^19^,^20^,^21^, the (forwards-direction) momentum should counteract the averaged position at offset (backwards direction). Therefore, the amount of perceptual shift at the onset position must be larger than that at the offset position. This momentum effect would also explain a little overshoot of the path of one-way motion at relatively slow speed: the temporal averaging view shows a little backwards shift at the offset, and therefore, the forwards shift due to motion representation might surpass the backwards shift. We must note that the task of a previous study^30^ was simply to adjust the length of the bar near the motion path to represent the perceived length of the path. Therefore, it is unclear how the motion representation would affect a positional shift at the endpoints derived from the temporal average of the instantaneous position. To confirm this, we must measure the positional shifts separately (independently) at the two endpoints.

Our primary objective was to investigate whether high-speed one-way moving objects can induce perceptual shrinkage by the method of constant stimuli (Fig. 3). Secondarily, we examined the effect of motion representation on the positional shift at the endpoints in the one-way motion path. In this study, we measured the perceptual positions of onset and offset of one-way motion. We deliberately manipulated the motion stimuli at a wide range of speeds (4–100 deg/s). The experimental results would reveal the integrated effect on perceptual shift of temporal averaging of spatial position and the motion representation. To display the motion stimuli at various speeds, we adopted a high-speed DLP projector. This enabled presentation of pseudo-realistic continuous motion, even in the high-speed conditions. By means of this display device, we overcame the limitations of the conventional CRT display and LCD, with which a large spatial gap would be inevitably inserted between images (or frames) used to create high-speed motion.

## Results

### Perceptual offset position

We calculated the proportion of trials that participants perceived that the test stimuli was shifted in the motion direction (forwards bias ratio: FBR), and then plotted its mean ratio as a function of the test position (Fig. 4). The data were pooled across left and right motions and the five participants. We omitted the data from one participant because he misunderstood the experimental instruction (i.e. he tracked the moving object with his eyes). The FBR shows that the perceptual shift was generally observed in the direction opposite that of the motion. The amount of the shift was larger for faster motion. We estimated the point of subjective equality of the perceived offset position (PSE) from the psychometric function of each participant by fitting a sigmoid function (“sigmoidal dose-response” of Prism 6, GraphPad Software inc.) (Fig. 4b). PSE ± SE averaged over participants were −0.15 ± 0.17 deg (4.0 deg/s), −0.28 ± 0.15 deg (8.3 deg/s), −0.31 ± 0.22 deg (16.7 deg/s), −0.47 ± 0.28 deg (33.3 deg/s), −0.91 ± 0.32 deg (66.7 deg/s), and −0.92 ± 0.30 deg (100.0 deg/s), indicating the larger backwards shift for faster speed. To evaluate the amount of the positional shift between speed conditions, we conducted one-way ANOVA. It revealed that the main effect of speed was significant (*F*_(5, 20)_ = 6.92, *p* < 0.001, *η*^2^ = 0.268). As for the main effect, Tukey’s multiple comparisons test revealed that the perceptual shifts of 66.7 and 100.0 deg/s were significantly greater than those of the 4.0, 8.3, and 16.7 deg/s conditions (all *ps* < 0.05). The other differences between conditions were not significant. We also conducted a one-sample two-tailed *t*-test to assess the difference between zero and the obtained PSEs. It revealed that the conditions 66.7 and 100.0 deg/s were significantly smaller than zero (4.0 deg/s: *t*(4) = 0.90, *p* = 0.420, *Cohen’s d* = 0.64; 8.3 deg/s: *t*(4) = 1.94, *p* = 0.124, *Cohen’s d* = 1.37; 16.7 deg/s: *t*(4) = 1.40, *p* = 0.235, *Cohen’s d* = 0.99; 33.3 deg/s: *t*(4) = 1.71, *p* = 0.163, *Cohen’s d* = 1.21; 66.7 deg/s: *t*(4) = 2.84, *p* = 0.047, *Cohen’s d* = 2.01; 100 deg/s: *t*(4) = 3.01, *p* = 0.040, *Cohen’s d* = 2.13).

### Perceptual onset position

The mean FBR at motion onset was also calculated in the same manner (Fig. 5). The results show that the perceptual onset position was shifted in the forwards direction, consistent with previous reports (Fröhlich effect^34^,^35^,^36^) and the spatial averaging view. We also obtained a PSE of the onset position separately for different speed conditions. Note that data from one participant was discarded for the same reason as described above. The averaged PSE shows larger positional shifts for faster speed conditions: averaged PSE ± SE across participants were 0.61 ± 0.17 deg (4.0 deg/s), 0.81 ± 0.22 deg (8.3 deg/s), 1.09 ± 0.21 deg (16.7 deg/s), 1.43 ± 0.28 deg (33.3 deg/s), 1.89 ± 0.55 deg (66.7 deg/s), and 1.52 ± 0.30 deg (100.0 deg/s). One-way ANOVA revealed that the main effect of speed was significant (*F*_(5, 20)_ = 6.16, *p* = 0.001, *η*^2^ = 0.326). As for the main effect, Tukey’s multiple comparisons test revealed that the perceptual shift of 66.7 deg/s were significantly greater than those of 4.0 deg/s and 8.3 deg/s conditions, and the perceptual shift of 100.0 deg/s was significantly greater that that of 4.0 deg/s (all *ps* < 0.05). The other differences between conditions were not significant. A one-sample two-tailed *t*-test for the difference between PSEs and veridical positions revealed that all speed conditions produced differences significantly larger than zero (4.0 deg/s: *t*(4) = 3.51, *p* = 0.025, *Cohen’s d* = 2.48; 8.3 deg/s: *t*(4) = 3.76, *p* = 0.020, *Cohen’s d* = 2.66; 16.7 deg/s: *t*(4) = 5.23, *p* = 0.006, *Cohen’s d* = 3.70; 33.3 deg/s: *t*(4) = 5.04, *p* = 0.007, *Cohen’s d* = 3.56; 66.7 deg/s: *t*(4) = 3.45, *p* = 0.026, *Cohen’s d* = 2.44; 100.0 deg/s: *t*(4) = 5.11, *p* = 0.007, *Cohen’s d* = 3.61).

### Difference between offset and onset positions

To compare the effects of speed and the differences of absolute shift at endpoints (i.e. onset vs. offset) on perceptual positional shifts, we conducted a mixed-design ANOVA for these PSEs. It revealed that the main effect of motion speed was significant (*F*_(5, 40)_ = 9.72, *p* < 0.001, *η*^2^ = 0.208), the main effect of position difference (*F*_(1, 8)_ = 4.15, *p* = 0.076, *η*^2^ = 0.203), and the interaction were not significant (*F*_(5, 40)_ = 1.28, *p* = 0.292, *η*^2^ = 0.027). As for the main effect of the speed condition, Tukey’s multiple comparisons test revealed that the PSEs of 33.3, 66.7 and 100.0 deg/s were significantly greater than that of the 4.0 deg/s condition. It also showed that the PSEs of 66.7 and 100.0 deg/s were significantly greater than those of the 8.3 deg/s condition, and that of 66.7 deg/s was significantly greater than that of 16.7 deg/s ccondition (all *ps* < 0.05). The other PSE differences between speed conditions were not significant. These results suggest that higher-speed motion robustly induces the positional shift, and that the amount of this shift tended to be greater at the onset position than at the offset position.

## Discussion

**Figure 2:**
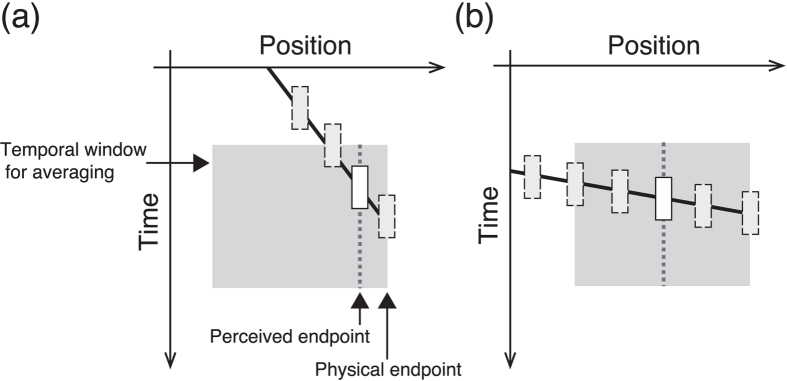
Perceptional shift of the offset position of a one-way moving object and its speed dependency. (**a**) When the speed is slow, the perceptual offset position should be close to the physical offset position because of the limited temporal range for averaging. (**b**) Discrepancy between the perceptual and physical offset positions should become greater at higher speeds.

Our results clearly demonstrate that high-speed motion can cause perceptual shrinkage of the one-way motion path: both endpoints of the motion path were perceived as shifted toward the centre of the path. These characteristics were consistent with our hypothesis that the shrinkage of one-way motion can be explained by the temporal averaging view (Fig. 2). The onset position was perceived as shifted in the forwards direction, identical to the shift observed in the Fröhlich effect^34^,^35^,^36^. This can be explained by temporal integration of the sequential positional signals^11^,^37^. The offset position, however, was perceived as shifted backwards, not in the direction of motion momentum, and/or a typical positional shift^19^,^20^,^21^: perceptual shrinkage of the one-way motion path therefore should not simply consist of forwards positional shifts of motion. Also, in the present study, the positional shifts were observed without any transient cue^10^,^11^,^12^,^15^, indicating the shifts are independent of other temporal cueing; i.e. it is plausible that shrinkage of the path is mainly induced by the motion stimulus itself. We also showed that the perceptual shift was significantly larger when the speed of motion was faster (Figs 4 and 5), and that this characteristic was observed at both onset and offset positions. This speed dependency robustly supports the temporal averaging view (Fig. 2).

Given that the perceived position would simply represent temporal averaging, the amount of positional shift in the faster speed condition suggests a minimal window width of the temporal average. For example, when a 100-ms averaging widow is applied to the moving object at 100 deg/s, it goes through the 10-deg motion path within that temporal window; therefore, the resulting average position should be 5 deg away from the endpoint (cf. Fig. 2). Following this, a 40-ms widow width induces a 2-deg positional shift (because it goes through a 4-deg motion path). In the same way, a 90-ms averaging widow for the 33.3 deg/s condition causes a 1.5-deg positional shift. The 2-deg and 1.5-deg shifts are close to the onset positional shifts observed in the 100.0 and 33.3 deg/s conditions, respectively. In contrast, as described previously, we found a speed dependency of the positional shift, whilst the amount of positional shift did not differ significantly in the higher-speed motion conditions (33.3, 66.7, and 100.0 deg/s). The results suggest a possible ceiling effect for faster speed conditions, because the positional shift increased almost logarithmically (Figs 4 and 5). Although this may be related to a saturation or a ceiling effect of the motion detection mechanism^38^,^39^,^40^,^41^, limitation on the spatial range for temporal averaging is another possible reason. We could not draw any conclusions about the precise range for inducing the perceptual shift at the endpoints, but our results seem to indicate that the temporal limit of the average might range from about 40 to 90 ms. This particular temporal range is not inconsistent with that reported in a previous study (less than 80 ms)^11^. According to the assumed temporal range, the spatial limit of the average may be about 3 or 4 deg. The range of the spatiotemporal average might be revealed in detail if even faster stimuli (>100.0 deg/s) were presented on a larger display than the one we used. In any case, our results are generally consistent with the temporal averaging view in respect to the speed dependency and the backwards shift at offset position.

Note that although the statistical difference was only at a marginally significant level, the perceptual shifts were consistently larger at the onset than at the offset position. This asymmetry cannot be explained simply by the temporal averaging view. One possible reason for this asymmetry is a difference in attentional state between onset and offset positions. A previous study^36^ reported that the size of the Fröhlich effect was decreased when attention was drawn near the onset position by a valid cue, although another study failed to show this cueing effect: the size of Fröhlich effect was not different with a transient cue and with a static landmark as a reference for the onset position^11^. In addition, the earlier part of the motion path would be an automatic cue to draw attention to the later part of the path^42^. Because our moving stimuli were presented without any cue, attention was not drawn to the onset position, whilst at the offset position, attention might be drawn by observation of the whole motion path. Such an attentional difference might cause the slight differences observed between the onset and offset of the motion.

Another possible cause is the difference in motion momentum between the two endpoints. The amount of positional shift depended mainly on the motion speed (Fig. 2). If the amounts of positional shift are different even within the same speed conditions (as we observed in onset and offset positions), it is reasonable to think that the difference derives from some factors related to the motion because the spatial average should be the same. Therefore, we assume that the amount of perceptual shift is determined by the “motion momentum”, that is, the continuity of the motion signal between endpoints.

Assume that the perceptual position is produced by the integration of the instantaneous position representation and the motion representation. In addition, consider that the motion representation is more effective around the offset position compared with the onset position. This is because the motion representation in the visual system must be gradually established along the movement path: it is an empirical fact that human observer can perceive a abruptly presented “line” stimulus as an elongating line segment when an attentional cue is presented at its endpoint (i.e., “illusory line motion” effect)^43^, and the activity of visual cortex of an anaesthetized cat suggested that neural substrates for such a perceptual effect is a temporally sequential response along the motion path^44^. This being the case, at the onset position, the perceptual position would mainly depend on the average of the instantaneous positions because the motion representation has not been established sufficiently. Conversely, at the offset position, the average position and motion signals would counterbalance each other, though imperfectly. The motion momentum (forwards direction) does not completely cancel the positional shift by the temporal average of instantaneous position (backwards direction). This is because the resulting perceived position is not consistent with the veridical position, but the robust backwards shifts were observed especially in higher-speed conditions (Fig. 4).

Before concluding, we would like to note that a high-speed DLP projector was essential for the present study. This can present moving and/or flicker (binary) images at a maximal 5000 Hz refresh rate, realizing continuous (i.e. not spatiotemporally-sparse) motion even in high-speed motion, which is impossible with the conventional CRT and LC displays. The authors expect that this device will promote further investigation of high-speed visual processing, which could not be performed in the past because of the technical limitation.

In conclusion, our results clearly demonstrate that one-way motion can induce shrinkage of the motion path for a high-speed moving object (at least 33.3 deg/s). The shrinkage was caused at both endpoints: onset position was perceived as shifted in the forwards direction, whilst offset position was perceived as shifted backwards. The amount of positional shift was highly dependent on motion speed and marginally dependent on the endpoint (i.e. onset vs. offset). We have argued that these results can be explained consistently by the view that the positional coding is derived from the integration of the spatiotemporal average of instantaneous positional information and the motion momentum: positional coding that is calculated by the temporal average could be modulated by the sequential motion signal, i.e. motion momentum.

## Methods

### Measurement for perceptual offset position

#### Apparatus

Stimuli were projected onto a distal screen (width 87 cm × height 66 cm: width 25 deg × height 19 deg) using a high-speed DLP LED projector (DLP Light Commander, Texas Instruments, Inc. Dallas, TX, spatial resolution: 748 × 567 pixels) in a dark room. The projector’s refresh rate was changed depending on the speed condition (described in detail herewith). Participants sat in a chair, and a viewing distance (202 cm) was held constant by a chin rest. Eye movements were not monitored. The projector was controlled by the API for the projector (distributed by Texas Instruments, Inc.) and Psychlops software (http://psychlops.osdn.jp/ja/)^45^ on a MacBook Pro (Apple, Inc. Cupertino, CA, USA). Due to technical limitations of the projector, the stimulus images used white and black luminances, i.e. 1-bit bitmap images. The same setup has also been used elsewhere^46^.

#### Stimuli

**Figure 3:**
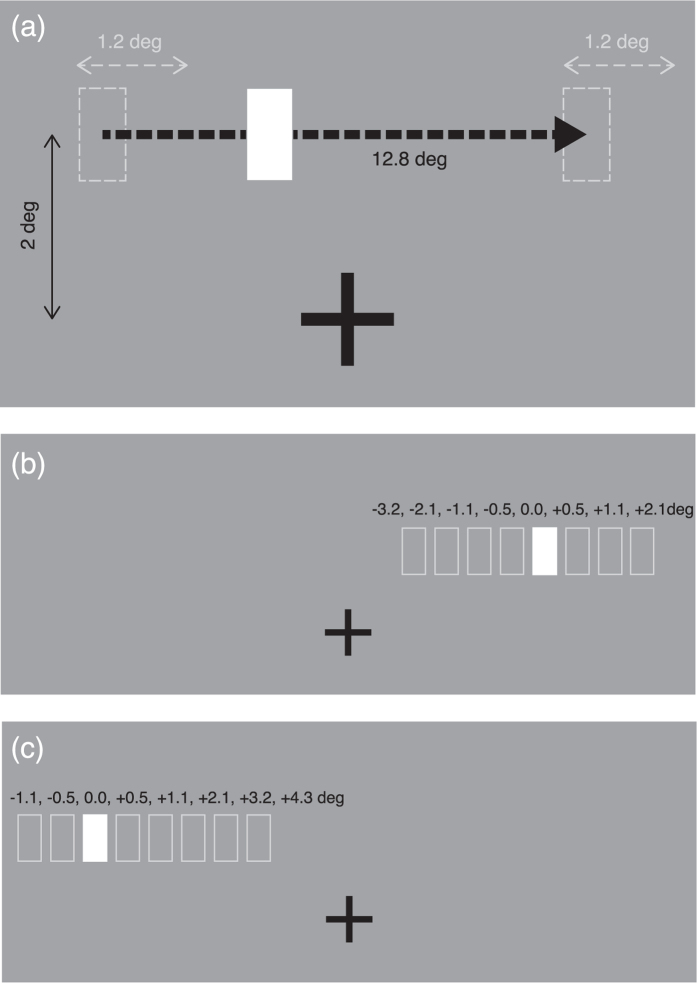
Schematic diagrams of the stimuli. (**a**) A white rectangle moved along a one-way horizontal path at various speeds (4.0 deg/s–100.0 deg/s). The path length was fixed at 12.8 deg, whilst position was randomly determined within a range of 1.2 deg (grey dashed arrows). The object appeared transiently and disappeared as soon as it arrived at the offset position. (**b**) After the object disappeared, a test stimulus (a white rectangle) appeared near the veridical offset position for measurement of perceptual offset position. Position of the test stimulus was chosen randomly from eight positions between −3.2 deg and +2.1 deg (rectangular frames). (**c**) Alternative positions of the test stimulus for measurement of perceptual onset position.

A white, vertical rectangle (0.5 deg × 1.0 deg: 15 pixels × 30 pixels, 19.5 cd/m^2^) moved in the horizontal (leftward or rightward) direction. The length of the motion path was 12.8 deg (384 pixels). The path was located 2 deg above the centre of a fixation cross that appeared at the centre of the screen. To prevent the participants from memorizing the endpoints of the motion path, the onset position of motion was randomly assigned within a range of 1.2 deg from trial to trial. The resulting offset position ranged between 4.8 deg (144 pixels) to 6 deg (180 pixels) from the centre of the screen. The background was filled with a 1-pixel checkerboard pattern to simulate the middle grey luminance of the projector (Fig. 3a).

#### Procedure

In a trial, a 300-ms blank frame (background with the fixation cross) appeared, after which the fixation cross remained on the screen as a vertical rectangle appeared transiently and moved in either the leftward or rightward direction. After the rectangle finished moving and disappeared, the blank frame was presented again for 500 ms and another vertical rectangle (the test stimulus) was shown near the offset position of the moving rectangle. The test stimulus was presented at either −3.2, −2.1, −1.1, −0.5, 0.0, +0.5, +1.1, or +2.1 deg, where 0.0 indicates the veridical offset (disappeared) position, and a positive (negative) value indicates that the test stimulus was displaced forwards (backwards) of the direction of motion (Fig. 3b). The spatial range of the test stimuli was determined by our preliminary observations. The motion direction and position of the test stimulus were randomly selected. Participants were asked to keep fixating on the centre cross during a trial, and to answer whether the test stimulus was located leftward or rightward relative to the perceived offset position of the moving rectangle. The response was converted into either a forwards or backwards value depending on the motion direction, e.g. when the object moved in the leftward direction and the answer was “leftward”, the response was converted to “forwards”. The test stimulus remained visible until a response was made.

In a trial block, speed of the moving object was randomly assigned as 4.0, 8.3, 16.7, 33.3, 66.7, or 100.0 deg/s. To equalize the spatial distance between frames, the speed was always 2 pixels/frame, and total number of frames presented was 192 in every speed condition. We changed the refresh rate of the projector to manipulate the speed (60, 125, 250, 500, 1000, or 1500 Hz, respectively). Therefore, the stimulus durations in the speed conditions were 3200, 1536, 768, 384, 192, and 128 ms, respectively. During a trial block, each of the 16 experimental conditions (2 directions × 8 test stimuli) was presented randomly five times. The total number of blocks was 12 (6 speeds × 2 repetitions).

#### Participants

Six males (mean ± SD age: 25.2 ± 4.9 years; range: 22–36 years) from the University of Electro-Communications participated in this experiment. One of them was author YN. No participants had any neurological or visual disorders, and all had normal or corrected-to-normal vision. All participants gave their written informed consent and all experimental protocols were reviewed and approved by the ethics committee of the University of Electro-Communications. All experiments were performed in accordance with the approved guidelines.

### Measurement for perceptual onset position

**Figure 4:**
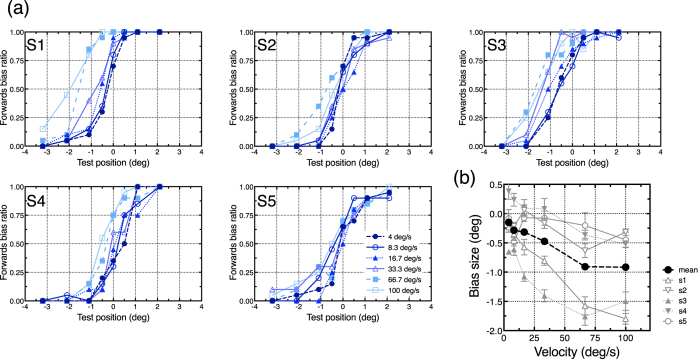
The results of perceptual offset measurement. (**a**) Forwards bias ratios (FBR) of five participants are plotted as a function of the positions of test stimuli. Each data point was obtained from a total of 20 trials. The zero on the x-axis indicates the veridical offset position of the moving object. (**b**) PSE of the offset position for each participant (grey lines) and average PSE across participants (black dashed line) were obtained. The error bars indicate the standard error from each participant’s fitting curve. The horizontal axis indicates the speed of moving objects and the vertical axis denotes the amount of the perceptual shift (bias size).

**Figure 5:**
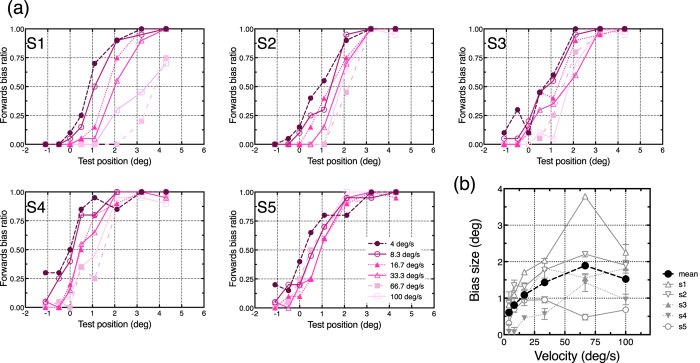
The results of perceptual onset measurement. The data in (**a**) and (**b**) are plotted in the same manner as in Fig. 4.

Apparatus, stimuli, procedure and participants were the same as for measurement for perceptual offset position, except for the following points: two females and four males (mean ± SD age: 24.5 ± 5.4 years; range: 20–36 years) in the University of Electro-Communications participated in this experiment. Three of them were also participated in the experiment for measuring the perceptual offset position. The test stimulus was presented at −1.1, −0.5, 0.0, +0.5, +1.1, +2.1, +3.2, or +4.3 deg relative to the veridical onset (presented) position, where a positive (negative) value indicates that the test stimulus was located in the forwards (backwards) motion direction (Fig. 4). To avoid participants memorizing the length of the motion path, the onset position of the moving stimulus was assigned randomly within 1.2 deg from trial to trial. Resulting onset position ranged between 4.7 deg (140 pixels) and 5.9 deg (176 pixels) from the centre of the screen, corresponding closely to the eccentricity of test position in the measurement for perceptual offset position.

## Additional Information

**How to cite this article**: Nakajima, Y. and Sakaguchi, Y. Perceptual shrinkage of a one-way motion path with high-speed motion. *Sci. Rep.* **6**, 30592; doi: 10.1038/srep30592 (2016).

## Figures and Tables

**Figure 1:**
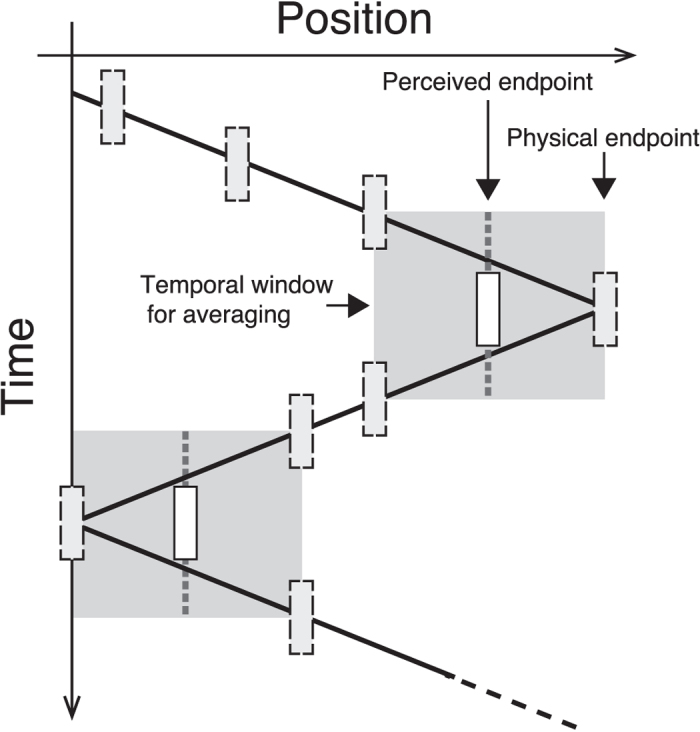
Temporal averaging model of position perception for a moving object. The solid zig-zag line represents the spatiotemporal trajectory of the object (light greyish rectangles). This model hypothesizes that the perceived position of the object is determined by the spatiotemporal average of object position within a certain time window (grey fields). Eventually, the perceived endpoints were shifted towards the centre of the motion path (white rectangles).
